# Accuracy of a machine learning method based on structural and locational information from AlphaFold2 for predicting the pathogenicity of *TARDBP* and *FUS* gene variants in ALS

**DOI:** 10.1186/s12859-023-05338-5

**Published:** 2023-05-19

**Authors:** Yuya Hatano, Tomohiko Ishihara, Osamu Onodera

**Affiliations:** Department of Neurology, Brain Research Institute, Niigata University, 1-757 Asahimachidori, Chuo-ku, Niigata-shi, Niigata 951-8585 Japan

**Keywords:** MOVA, Missense variant, Prediction tool, Amyotrophic lateral sclerosis, AlphaFold2

## Abstract

**Background:**

In the sporadic form of amyotrophic lateral sclerosis (ALS), the pathogenicity of rare variants in the causative genes characterizing the familial form remains largely unknown. To predict the pathogenicity of such variants, in silico analysis is commonly used. In some ALS causative genes, the pathogenic variants are concentrated in specific regions, and the resulting alterations in protein structure are thought to significantly affect pathogenicity. However, existing methods have not taken this issue into account. To address this, we have developed a technique termed MOVA (method for evaluating the pathogenicity of missense variants using AlphaFold2), which applies positional information for structural variants predicted by AlphaFold2. Here we examined the utility of MOVA for analysis of several causative genes of ALS.

**Methods:**

We analyzed variants of 12 ALS-related genes (*TARDBP*, *FUS*, *SETX*, *TBK1*, *OPTN*, *SOD1*, *VCP*, *SQSTM1*, *ANG*, *UBQLN2*, *DCTN1*, and *CCNF*) and classified them as pathogenic or neutral. For each gene, the features of the variants, consisting of their positions in the 3D structure predicted by AlphaFold2, pLDDT score, and BLOSUM62 were trained into a random forest and evaluated by the stratified fivefold cross validation method. We compared how accurately MOVA predicted mutant pathogenicity with other in silico prediction methods and evaluated the prediction accuracy at *TARDBP* and *FUS* hotspots. We also examined which of the MOVA features had the greatest impact on pathogenicity discrimination.

**Results:**

MOVA yielded useful results (AUC ≥ 0.70) for *TARDBP*, *FUS*, *SOD1*, *VCP*, and *UBQLN2* of 12 ALS causative genes. In addition, when comparing the prediction accuracy with other in silico prediction methods, MOVA obtained the best results among those compared for *TARDBP*, *VCP*, *UBQLN2*, and *CCNF*. MOVA demonstrated superior predictive accuracy for the pathogenicity of mutations at hotspots of *TARDBP* and *FUS*. Moreover, higher accuracy was achieved by combining MOVA with REVEL or CADD. Among the features of MOVA, the x, y, and z coordinates performed the best and were highly correlated with MOVA.

**Conclusions:**

MOVA is useful for predicting the virulence of rare variants in which they are concentrated at specific structural sites, and for use in combination with other prediction methods.

**Supplementary Information:**

The online version contains supplementary material available at 10.1186/s12859-023-05338-5.

## Introduction

Rare variants in the causative genes of familial amyotrophic lateral sclerosis (FALS) are found in 10–30% of cases of sporadic ALS (SALS) [5, 7]. However, the pathological significance of such variants occurring only in SALS is largely unknown. The pathogenicity of these rare variants must be validated using cultured cells, animal models, and samples obtained from patients. However, this type of validation is generally difficult to perform. Instead, in silico analytical methods can be used to predict pathogenicity [1, 8, 9, 15, 18].

In silico analysis focuses primarily on evolutionary conservation of gene and amino acid similarity; the most commonly used algorithm is PolyPhen-2, which is a machine learning approach employing eight variables based on nucleotide sequences and variables predicted from known three-dimensional (3D) structures [1]. REVEL and CADD are methods that integrate several in silico analysis methods, including PolyPhen-2 [9, 15]. EVE, a machine-learned method utilizing evolutionary conservation of sequences, has also been reported to predict the pathogenicity of rare variants without supervised data [8]. Neither method alone is recommended for identifying pathogenicity. Guidelines for genetic diagnosis from the American College of Medical Genetics and Genomics (ACMG) and the Association for Molecular Pathology (AMP) recommend that these analytical methods should be used only when multiple methods predict that the variant is deleterious [16]. Therefore, development of a new in silico approach from a new perspective would be desirable.

One factor not often considered in existing in silico analysis methods is the location of the variant and the 3D structure of the associated region [18]. In the ALS-associated genes *TARDBP* and *FUS*, mutations are concentrated in a particular region [3, 12]. These regions are also structurally characteristic and are assumed to undergo facilitated aggregation. If positional information and pathogenicity related to the structure of these regions can be taken into account, more accurate estimation of pathogenicity might be possible. Indeed, the ACMG guidelines recommend that even if a variant is located in a mutational hotspot or in an important functional domain, it is a factor that would support the pathogenicity of the variant [16]. Therefore, the accuracy of pathogenicity prediction would be improved by taking into account positional information about the variants. However, it has been difficult to utilize this factor because of the limited number of proteins whose 3D structures have been clarified [18].

Recently, AlphaFold2 was developed as a method for prediction of protein 3D structures in silico with high accuracy [10]. This method can predict the structure of a protein even in the absence of a similar protein whose structure is already known [17]. Therefore, addition of structural information using AlphaFold2 would be expected to improve the accuracy of pathogenicity prediction. In fact, AlphScore has been shown to predict the pathogenicity of rare variants using this factor [18], and when combined with REVEL, CADD, and DEOGEN2, it improved the accuracy of each program [18]. These results suggest that structural information from AlphaFold2 would be useful for pathogenicity prediction.

Here we developed an approach termed MOVA (a method for evaluating the pathogenicity of missense variants using AlphaFold2), which applies positional information for variants based on the 3D structure predicted by AlphaFold2, and machine-learns the pathogenicity of variants for each gene. We investigated the usefulness of MOVA for predicting the pathogenicity of rare ALS-causative gene variants.

## Materials and methods

### Gene set

We counted the types of ALS-causing mutations in known ALS-causing genes using HGMD Professional 2022.4, and the top 12 genes with the most types of causative mutations, *SOD1*, *TARDBP*, *FUS*, *VCP*, *SETX*, *TBK1*, *SQSTM1*, *ANG*, *OPTN*, *UBQLN2*, *DCTN1*, and *CCNF* were included in the analysis (Additional file 1: Table S1). Mutation of the ALS causative gene *C9orf72* was excluded from this analysis because of a repeat extension mutation.

### Data set

Variants of each gene listed in gnomAD v.3.1.2 or HGMD Professional 2022.4 were included in the analysis. Pathogenic variants (positive variants) were defined as variant class ‘DM’ with reported phenotypes including amyotrophic lateral sclerosis, frontotemporal dementia, or motor neuron disease in HGMD Professional 2022.4. Neutral variants (negative variants) were defined as variants recognized in gnomAD v.3.1.2 excluding variants defined as a class ‘DM’ or ‘DM?’ in HGMD Professional 2022.4.

### CADD, PolyPhen-2, EVE, REVEL and AlphScore

We evaluated the accuracy with which CADD, PolyPhen-2, EVE, REVEL, and AlphScore were able to discriminate between positive and negative variants in the existing datasets. PolyPhen-2 used HumDiv as the classifier model. Sensitivity (true-positive rate) versus 1-specificity (false-positive rate) at each threshold was plotted as a ROC curve, and the AUC was calculated. The specificity and sensitivity at each threshold were calculated using the prediction function of the ROCR package in R version 4.2.2, and the ROC curve plot and AUC were calculated using the performance function.

### MOVA: a method for determining variant pathogenicity using AlphaFold2

**Fig. 1:**
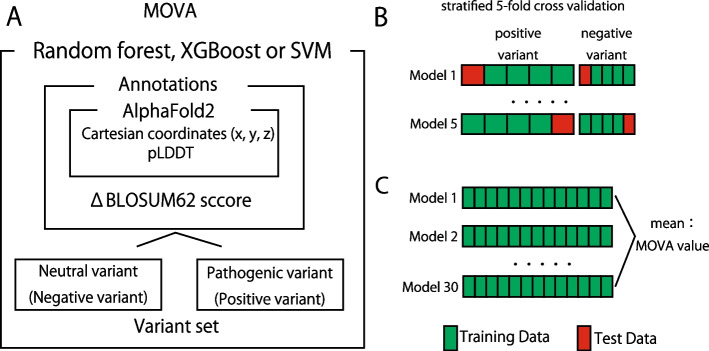
Work flowchart for MOVA. The x, y, z coordinates, and the plddt score for the amino acid residues at the substitution sites in the protein in the pdb file of the Alphafold2 database, and the ΔBLOSUM62 of the substituted amino acid residue, were used as parameters for random forest, XGBoost, or support vector machine (SVM) training (**A**). The sample group was randomly divided into five subsets as avoiding bias in objective variables. With one subset as the test cases and the rest as the training cases, we built the model. The predictions were calculated and validated using the test data. The models were iteratively built so that all five subsets were test cases. (**B**). The model was generated 30 times with all variants in the dataset as training data. The probability of each possible variant of the gene being pathogenic was predicted, and the average of the predictions was used as the MOVA value (**C**)

To construct MOVA, we used supervised machine learning with labeled examples attributed to positive or negative variants, and trained and examined each gene individually. The average x, y and z coordinates for each atom and the pLDDT (predicted local distance difference test) score at the site of the mutant amino acid residue of the protein in the pdb file of the Alphafold2 database [19], and “the likelihood of an event in which the reference amino acid at the site of amino acid change remains the same—the likelihood of an event in which the reference amino acid is replaced by the alternative amino acid, as evaluated by BLOSUM62” were used as features. They were trained with random forest, Support Vector Machine (SVM) or XGBoost and evaluated using the stratified fivefold cross validation method [4, 6, 11]. These steps were repeated five more times to reduce the variation from one execution to the next. Finally, we considered the method with the highest mean AUC for the 12 target genes among the three methods (Fig. 1A). The random forest model was constructed using the randomForest function from the randomForest package of the statistical software R version 4.2.2, the SVM model was constructed using the ksvm function from the kernlab package, and the XGBoost model was constructed using the xgb.train function from the xgboost package. Parameters for random forest and SVM were left at the default settings of the randomForest function and ksvm function, XGBoost parameters were set to objective = “binary:logistic”, booster = “gbtree”, nrounds = 100, and all other parameters were left at their default settings.

#### Comparison of MOVA with the models based on each of MOVA's features

We compared the usefulness of MOVA to models that use each of the features that constitute MOVA. The average x, y, and z coordinates of each atom at the site of the mutated amino acid residue of the wild-type protein in the AlphaFold2 database were used as the feature values, trained in a random forest, and evaluated using a stratified fivefold cross-validation method. To reduce variation from one execution to the next, they were repeated five more times and compared to MOVA. Similarly, the average of pLDDT scores alone, ΔBLOSUM62 alone, and the average of pLDDT scores and ΔBLOSUM62 excluding x, y, and z coordinates as features were trained using the random forest method, evaluated using the stratified fivefold cross-validation method, and their usefulness was compared with MOVA.

#### The simple approach based on distance from known pathogenic variants

We used the stratified fivefold cross-validation method to evaluate whether pathogenicity could be predicted by the distance between the target variant and a known pathogenic variant. Following the stratified fivefold cross-validation method, we divided the training and test data and calculated the distance in 3D position calculated by AlphaFold2 for a variant of test data for each pathogenic variant of the training data. The minimum distance was taken as the 'distance to known pathogenic variants.’ The stratified fivefold cross-validation method was repeated five more times to reduce the variation from run to run. The AUC was used to evaluate whether the positive or negative variant could be distinguished from the ‘distance from known pathogenic variants’ for the test cases. The ROC curve plot and AUC were calculated using the cvAUC function of the cvAUC package in R, version 4.2.2.

### MOVA model evaluation

In this analysis, the model was evaluated using the stratified fivefold cross-validation method because of the bias in the number of pathogenic mutations and neutral polymorphisms in the data set (Fig. 1B). The sample group was randomly divided into five subsets as avoiding bias in objective variables. We made it one-fold to build the model with one subset as test data and the rest as training data. Five folds were repeated so that all five subsets were the test data. We repeated this process five more times to reduce the variability from one execution to the next. Predicted values were calculated using the prediction function of the randomForest package in R version 4.2.2, with the average being the 5F-MV value:fivefold MOVA value. Plotting of ROC curves and calculation of the AUC were performed using the cvAUC function of the cvAUC package. The specificity and sensitivity at each threshold for each fold were calculated using the prediction function of the ROCR package. The cutoff value was set to the mean of the Youden index (the point at which sensitivity + specificity − 1 is the maximum value).

### Combination with other missense prediction scores

MOVA was combined with REVEL or CADD using logistic regression as implemented in the R-function glm with the option family = binomial in R version 4.2.2 and evaluated using the stratified fivefold cross validation method. The combination of AlphScore with REVEL or CADD was used directly from https://zenodo.org/record/6288139#.Ym0Ir9rP23A.

### Generation of the final MOVA model

The predicted value of the probability of each variant being pathogenic was calculated by MOVA, and this was used as the MOVA value. The model was fitted using all the variants in the dataset as training data. Since machine learning can change values from each trial, the model was generated in a prediction trial of 30 times and the average of these predictions was used as the MOVA value (Fig. 1C). The value was set to 0 if the allele was the same as the reference. MOVA values range from 0 to 1, with 1 having the highest probability of being pathogenic. These analyses were performed using the statistical software R version 4.2.2 (Additional files 2–13: Tables S2–S13).

### Spearman’s rank correlation coefficient

Spearman’s rank correlation coefficient was performed to observe the relationship between the AUC of MOVA and the number of variants in the dataset. In addition, the correlation between the AUC of MOVA and the AUC of each feature-specific analysis was also evaluated using Spearman's rank correlation coefficient. The analysis was performed using the statistical software R version 4.2.2.

## Results

Figure 1 shows how MOVA was set up. We assumed that the location of the protein mutation affects pathogenicity and incorporated mainly those that conform to positional information as features. Among the features used, (1) the x, y, and z coordinates of the mutated amino acid residue in the 3D structure of the wild-type protein predicted by AlphaFold2 are the location information. (2) The pLDDT score was incorporated as a reliability score for location prediction. (3) BLOSUM62, which is also used in known pathogenicity prediction algorithms, was used to evaluate the evolutionary conservation of amino acids. In the present study, we did not use other protein structure-specific features because we emphasized compliance with protein location information. These data were used for training for each gene employing the random forest or SVM or XGBoost method. After the study, AUCs were calculated using a stratified fivefold cross-validation method to compare the three methods (Additional file 14: Table S14). In this study, an AUC of 0.70 or higher was considered a useful result [2].

There were 5 genes (*TARDBP*, *FUS*, *SOD1*, *VCP*, *UBQLN2*) out of 12 genes with AUC ≥ 0.70 in both random forest, XGBoost, and SVM. The mean AUC was 0.655 for the random forest, 0.645 for XGBoost, and 0.648 for SVM. We chose to continue the analysis with the random forest with the highest mean AUC.

**Fig. 2:**
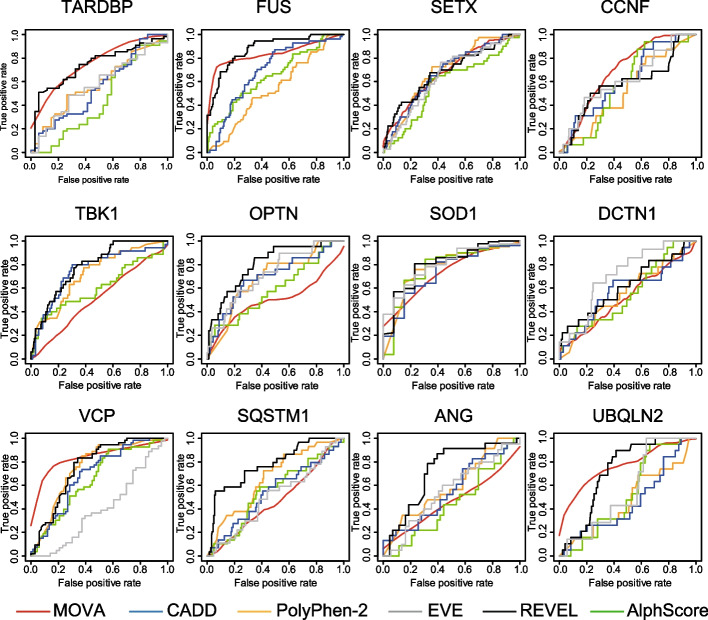
We used receiver operating characteristic (ROC) curve analysis to determine whether MOVA (red line), CADD (blue line), PolyPhen-2 (orange line), EVE (gray line), REVEL (black line), or AlphScore (green line) classified variants for *TARDBP*, *FUS*, *SETX*, *TBK1*, *OPTN*, *SOD1*, *VCP*, *SQSTM1*, *ANG*, *UBQLN2*, *DCTN1*, and *CCNF* as positive or negative. For MOVA, the stratified fivefold cross-validation was repeated 5 times, so the cvAUC function of the cvAUC package was used to draw the average of the ROC curves for 25 times

The cutoff values and AUCs for each gene in MOVA, as well as the number of pathogenic mutations and neutral polymorphisms used in the study, are summarized in Table 1. For the other in silico prediction methods, AUCs were calculated from the pre-computed predictions and compared with MOVA trained in the random forest (Table 2, Fig. 2).

**Table 1:** Performance of MOVA for the 12 ALS causative genes

Gene	AUC	Cutoff	Positive variant	Negative variant
*TARDBP*	0.772	0.63	55	34
*FUS*	0.841	0.429	54	153
*SETX*	0.678	0.0434	40	939
*TBK1*	0.513	0.153	35	153
*OPTN*	0.501	0.192	21	173
*SOD1*	0.786	0.861	181	13
*VCP*	0.847	0.509	53	67
*SQSTM1*	0.473	0.123	29	182
*ANG*	0.464	0.407	23	43
*UBQLN2*	0.769	0.279	19	118
*DCTN1*	0.524	0.124	18	390
*CCNF*	0.696	0.046	16	297

*AUC* area under the curve

**Table 2:** Performance of MOVA and the existing methods

gene	MOVA	PolyPhen-2	CADD	REVEL	EVE	AlphScore
*TARDBP*	0.772	0.583	0.555	0.742	0.571	0.448
*FUS*	0.841	0.541	0.694	0.878	–	0.652
*SETX*	0.678	0.692	0.675	0.682	0.649	0.577
*TBK1*	0.513	0.721	0.748	0.785	–	0.603
*OPTN*	0.501	0.686	0.693	0.804	0.72	0.589
*SOD1*	0.786	0.774	0.737	0.805	0.8	0.771
*VCP*	0.847	0.753	0.692	0.759	0.403	0.654
*SQSTM1*	0.473	0.661	0.541	0.775	0.504	0.548
*ANG*	0.464	0.593	0.555	0.721	0.554	0.443
*UBQLN2*	0.769	0.47	0.464	0.722	0.562	0.527
*DCTN1*	0.524	0.558	0.577	0.617	0.709	0.553
*CCNF*	0.696	0.533	0.615	0.581	0.619	0.578
Average	0.655	0.63	0.629	0.739	0.609	0.579

MOVA showed AUC ≥ 0.70 for 5 of 12 genes (*TARDBP*, *FUS*, *SOD1*, *VCP*, *UBQLN2*). Three of the 12 genes (*TBK1*, *SOD1*, *and VCP*) in PolyPhen-2, two of the 12 genes (*TBK1*, *SOD1*) in CADD, nine of the 12 genes (*TARDBP*, *FUS*, *TBK1*, *OPTN*, *SOD1*, *VCP*, *SQSTM1*, *ANG*, *and UBQLN2*) in REVEL, and one of the 12 genes (*SOD1*) in AlphScore showed an AUC ≥ 0.70. In EVE, 3 (*OPTN, SOD1, DCTN1*) of the 10 genes (*TARDBP, SETX, OPTN, SOD1, VCP, SQSTM1, ANG, UBQLN2, DCTN1, CCNF*) listed in the database (https://evemodel.org/) showed an AUC of ≥ 0.70. *SETX* and *CCNF* did not show an AUC of ≥ 0.70 for any of the methods. Six genes (*FUS*, *TBK1*, *OPTN*, *SOD1*, *SQSTM1*, *and ANG*) showed the best AUC with REVEL, and four genes (*TARDBP*, *VCP*, *UBQLN2*, *and CCNF*) showed the best AUC with MOVA. PolyPhen-2 showed the highest AUC for *SETX* and EVE for *DCTN1*. The mean AUC for the 12 genes for each method was highest for REVEL at 0.739, followed by MOVA at 0.655, PolyPhen-2 at 0.630, CADD at 0.629, EVE at 0.609, and AlphScore at 0.579 (Table 2).

Next, we examined whether the addition of 'distance to known pathogenic variants,’ which is positional information about the protein 3D structure, could improve the accuracy of MOVA pathogenicity prediction. In the analysis with only ‘distance to known pathogenic variants’ as a feature, four of the 12 genes (*TARDBP*, *FUS*, *SOD1*, *and VCP*) showed AUCs ≥ 0.70, and the mean AUC for the 12 genes was 0.656, showing similar usefulness to MOVA (Additional file 15: Table S15). However, adding 'distance to known pathogenic variants' as one of the features of MOVA did not improve the number of genes with AUC ≥ 0.70, 4 out of 12 genes (*TARDBP*, *FUS*, *VCP*, *UBQLN2*), and the mean AUC of the 12 genes was 0.629, with no improvement (Additional file 15: Table S15).

In addition, to examine which features of MOVA affect the accuracy of MOVA predictions, AUC was calculated for each gene by random forest analysis for each feature (Additional file 16: Table S16). The genes with AUC ≥ 0.70 were two of 12 genes (*TARDBP*, *FUS*) for pLDDT and one of 12 genes (*UBQLN2*) for BLOSUM62, while four of 12 genes (*TARDBP*, *FUS*, *SOD1*, *VCP*) for x, y, z coordinates. The mean AUC for the 12 genes was the highest for the 3D structure position coordinates: pLDDT 0.597, BLOSUM62 0.567, and x, y, z coordinates 0.625. Spearman's correlation coefficients for the AUCs calculated for each and MOVA were pLDDT-MOVA 0.80, BLOSUM62-MOVA 0.46, and position information-MOVA 0.93, with the highest correlation coefficient between 3D structure position information and MOVA. In addition, the features of pLDDT and BLOSUM62, excluding location information, were trained and evaluated in a random forest and the AUC was calculated (Additional file 16: Table S16). Only two of the 12 genes (*FUS* and *SETX*) had AUCs ≥ 0.70, and the average AUC for the 12 genes was 0.603, which was lower than the 3D structural position information alone.

**Fig. 3:**
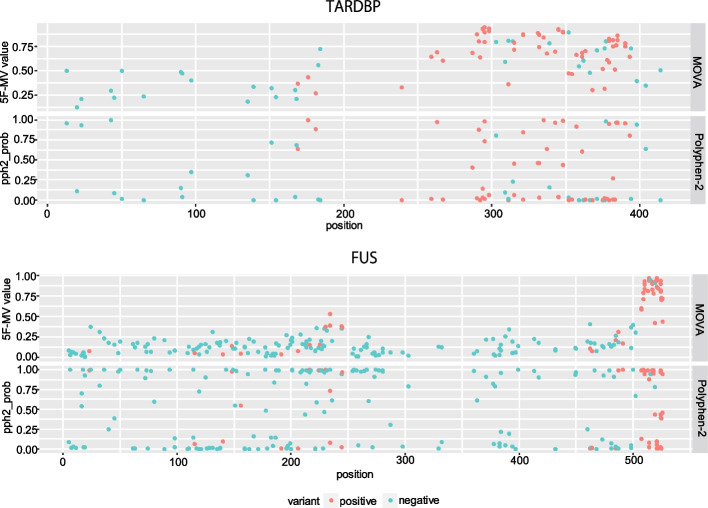
All variants of *TARDBP* and *FUS* in the dataset were divided into positive variants (red) associated with ALS and negative variants (light blue) recognized in the general population. For Polyphen-2, pph2_prob (classifier probability of the variation being damaging), and MOVA, the 5F-MV value was plotted on the y-axis as the predicted value and the residue number was plotted on the x-axis. Both pph2_prob and 5F-MV take values between 0 and 1, with 1 having the highest probability of being pathogenic

For *TARDBP* and *FUS*, the predicted probability of pathogenicity was compared between MOVA and PolyPhen-2 (Fig. 3). MOVA tended to have higher 5F-MV values at hotspots. In contrast, no such trend was observed for pph2_prob in Polyphen-2 (Fig. 3). MOVA values for each mutation in *TARDBP*, *FUS*, *SETX*, *TBK1*, *OPTN*, *SOD1*, *VCP*, *SQSTM1*, *ANG*, *UBQLN2*, *DCTN1*, and *CCNF* are shown in Additional files 2–13: Tables S2–S13.

Finally, we examined whether combining MOVA with CADD or REVEL could improve prediction accuracy. In CADD alone, only *TBK1* and *SOD1* showed AUC ≥ 0.7, but in CADD + MOVA, seven genes (*TARDBP*, *FUS*, *SETX*, *TBK1*, *SOD1*, *VCP*, *and UBQLN2*) showed AUC ≥ 0.70 (Table 3, Additional file 17: Fig. S1). The mean AUC for the 12 genes was higher for CADD + MOVA (0.690) than for CADD alone (0.629). Nine genes (*TARDBP*, *FUS*, *TBK1*, *OPTN*, *SOD1*, *VCP*, *SQSTM1*, *ANG*, and *UBQLN2*) showed AUC ≥ 0.7 in REVEL alone, while 10 genes, including SETX, showed AUC ≥ 0.7 in REVEL + MOVA (Table 3, Additional file 18: Fig. S2). The mean AUC for the 12 genes was also higher at 0.772 compared with 0.739 for REVEL alone.

**Table 3:** AUC for combination of MOVA and other methods

	MOVA	CADD			REVEL		
		Only	+ MOVA	+ AlphScore	Only	+ MOVA	+ AlphScore
*TARDBP*	0.772	0.555	0.765	0.533	0.742	0.795	0.709
*FUS*	0.841	0.694	0.837	0.737	0.878	0.903	0.888
*SETX*	0.678	0.675	0.71	0.659	0.682	0.712	0.682
*TBK1*	0.513	0.748	0.758	0.743	0.785	0.787	0.79
*OPTN*	0.501	0.693	0.667	0.703	0.804	0.812	0.797
*SOD1*	0.786	0.737	0.776	0.774	0.805	0.846	0.818
*VCP*	0.847	0.692	0.881	0.723	0.759	0.89	0.761
*SQSTM1*	0.473	0.541	0.47	0.55	0.775	0.753	0.766
*ANG*	0.464	0.555	0.516	0.542	0.721	0.716	0.692
*UBQLN2*	0.769	0.464	0.752	0.475	0.722	0.836	0.701
*DCTN1*	0.524	0.577	0.511	0.592	0.617	0.569	0.614
*CCNF*	0.696	0.615	0.641	0.617	0.581	0.646	0.613
Average	0.655	0.629	0.69	0.637	0.739	0.772	0.736

## Discussion

We developed a new in silico method for predicting the pathogenicity of missense variants, MOVA, based on the assumption that the position of the variant on the protein affects the pathogenicity. It was developed by machine learning using the pathogenicity information previously reported for each gene as training data, using the position of the mutation site on the 3D structure of each protein predicted by Alphafold2 as the feature value. Similarly, we compared the prediction accuracy of MOVA with that of AlphScore, which predicts pathogenicity using the three-dimensional structural data of AlphaFold2. When used for analysis of 12 ALS-causative genes, MOVA showed higher prediction accuracy than AlphScore for eight of them (*TARDBP*, *FUS*, *SETX*, *SOD1, VCP, ANG, UBQLN2 and CCNF*). Especially for *TARDBP* and *FUS*, MOVA was superior for determining pathogenicity at hotspots; MOVA performs machine learning on a gene-by-gene basis, which may explain its superior performance for predicting the pathogenicity of polymorphisms in pathogenic genes with hotspots.

We analyzed each feature in a random forest and calculated AUCs for each gene to examine whether the positional information of the variants on the protein influenced the good performance of MOVA. The results showed that the AUC of 3D structure-position coordinates was the highest and best correlated with MOVA, indicating that 3D structure-position coordinates contributed to the analysis of MOVA. We also tested whether 'distance to known pathogenic variants' can predict the pathogenicity of a variant. Although this also showed some usefulness, it did not contribute to improving the prediction accuracy of MOVA; therefore, it was not included in the MOVA features.

MOVA is a machine-learning method that focuses on specific genes. In this study, we analyzed 12 ALS-related genes and compared their effects with those of the existing in silico prediction methods. Overall, REVEL performed the best, followed by MOVA. However, each gene had a different algorithm that resulted in a favorable AUC. For a more accurate in silico analysis of the pathogenicity of novel genes, preliminary analysis using multiple algorithms, as was done in this study, would be necessary to select a useful algorithm. ACMG guidelines also support this idea, as it is important to use multiple in silico methods to predict pathogenicity [16]. Compared with existing algorithms, MOVA, which uses previously unused location information as the main analysis data, is a useful option.

AlphScore also increased accuracy when combined with CADD or REVEL, and MOVA, when combined with CADD or REVEL, showed AUCs that exceeded not only CADD and REVEL alone but also the combination of AlphScore and CADD or REVEL. These results demonstrate the usefulness of MOVA.

With regard to the characteristics of genes for which MOVA is effective, it is a machine-learning program that considers a variant’s position in the 3D structure predicted by AlphaFold2, the pLDDT score, which is the certainty of the prediction [10], and the likelihood of evolutionary change between the reference and the alternative amino acid residue derived from the BLOSUM62 score, using previously reported pathogenic mutation information as the teacher data. Therefore, it is effective when the pathogenicity depends on the position of the variant in the 3D structure and its localization to a specific region. If there is a hot spot of genetic variation, MOVA may increase the prediction accuracy. Indeed, in *TARDBP* and *FUS*, for which MOVA was effective, pathogenic mutations are concentrated at the C-terminus, and moreover, in *TARDBP*, they are concentrated in IDRs (intrinsic disordered regions) (Fig. 3, red circles) [3, 12]. Since pLDDT scores are associated with IDRs [14], MOVA may also be superior for detecting genes that are associated with IDRs and pathogenicity. However, in *TBK1* or *OPTN*, where the prediction accuracy of MOVA was low, there are no regions of concentrated pathogenic mutations [13, 20], suggesting that the characteristics of MOVA may not have been exploited.

The prediction accuracy of machine learning is known to depend on the number of teacher data. Two types of teacher data were used in this study: pathogenic and neutral variants. More than 50 pathogenic mutants were used for *TARDBP*, *FUS*, *SOD1*, and *VCP*, for which MOVA showed a high prediction accuracy. In contrast, 33, 21, 29, and 23 were used for *TBK1*, *OPTN*, *SQSTM1*, and *ANG*, respectively, which had low prediction accuracy. The number of pathogenic variants used for analysis in the MOVA may have affected the prediction accuracy; however, Spearman's rank correlation coefficient between the number of pathogenic variants used in the analysis and the MOVA AUC for each gene was 0.53 (*p* = 0.079), which was not a significant correlation. The number of neutral mutations was more than 100 for *FUS*, *SETX*, *TBK1*, *OPTN*, *SQSTM1*, *UBQLN2*, *DCTN1*, and *CCNF* and less than 100 for *TARDBP*, *SOD1*, *VCP*, and *ANG*; no association with MOVA prediction accuracy was found. Statistical analysis showed that the coefficient of Spearman’s rank correlation between the number of neutral variants used in the analysis and MOVA AUC was − 0.36 (*p* = 0.25).

One limitation of MOVA is that its results depend on the number of pathogenic variants used in the teacher data, so fewer teacher data will result in less accurate predictions. In our study, there were some genes for which MOVA was useful, even with as few as 15 pathogenic mutations, but it would not be used for genes with only three or four known pathogenic mutations. It cannot be used for genes with no known pathogenic mutations, such as newly identified disease-related genes. However, the amount of information on genetic variation is expected to increase further with the generalization of exome and whole-genome analyses. In this regard, the usefulness of MOVA is expected to increase. In addition, the data in this analysis were based on the location of the wild-type. For a more rigorous analysis, positional information must be calculated for all mutant proteins. However, it is too time-consuming and practically difficult to estimate the 3D structures of all possible mutant proteins; therefore, the analysis was based on the 3D structure of the wild type. Another limitation is that, compared to REVEL alone, MOVA has a lower overall performance. In this regard, rather than blindly using MOVA alone, it is necessary to devise ways to adapt MOVA to genes with high prediction accuracy, or to combine MOVA with other methods. Finally, only the ALS-related genes were included in this study. It remains to be seen whether the same usefulness of MOVA as in the present analysis can be demonstrated for causative genes of other diseases.

We have thus developed MOVA, a machine learning approach using the 3D structural position of a variant, as predicted by AlphaFold2, for learning and predicting whether or not a specific gene variant will be pathogenic. This method showed the second highest virulence discrimination rate after REVEL in the evaluation of 12 genes. For some genes, the discrimination rate of MOVA was higher than that of REVEL. This method is useful for predicting the pathogenicity of rare polymorphisms in genes in which mutations are clustered at specific structural sites. It also showed a high pathogenicity discrimination rate when combined with CADD and REVEL. This method will be useful for predicting the pathogenicity of rare gene variants in which mutations are clustered at specific structural sites.

## Supplementary Information

**Additional file 1: Table S1**. Number of ALS causative gene types.

**Additional file 2: Table S2**. MOVA final dataset in *TARDBP*.

**Additional file 3: Table S3**. MOVA final dataset in *FUS*.

**Additional file 4: Table S4**. MOVA final dataset in *SETX*.

**Additional file 5: Table S5**. MOVA final dataset in *TBK1*.

**Additional file 6: Table S6**. MOVA final dataset in *OPTN*.

**Additional file 7: Table S7**. MOVA final dataset in *SOD1*.

**Additional file 8: Table S8**. MOVA final dataset in *VCP*.

**Additional file 9: Table S9**. MOVA final dataset in *SQSTM1*.

**Additional file 10: Table S10**. MOVA final dataset in *ANG*.

**Additional file 11: Table S11**. MOVA final dataset in *UBQLN2*.

**Additional file 12: Table S12**. MOVA final dataset in *DCTN1*.

**Additional file 13: Table S13**. MOVA final dataset in *CCNF*.

**Additional file 14: Table S14**. Comparison of three machine learning methods.

**Additional file 15: Table S15**. AUC for MOVA and distance to known pathogenic mutations.

**Additional file 16: Table S16**. AUC for the individual features.

**Additional file 17: Figure S1**. We used receiver operating characteristiccurve analysis to determine whether MOVA + CADD, MOVA, CADD, or CADD + AlphScoreclassified variants for *TARDBP*, *FUS*, *SETX*, *TBK1*, *OPTN*, *SOD1*, *VCP*, *SQSTM1*, *ANG*, *UBQLN2*, *DCTN1*, and *CCNF* as positive and negative. For MOVA and MOVA + CADD, the stratified fivefold cross validation was repeated 5 times, so the cvAUC function of the cvAUC package was used to draw the average of the ROC curves for 25 times.

**Additional file 18: Figure S2**. We used receiver operating characteristiccurve analysis to determine whether MOVA + REVEL, MOVA, REVEL, or REVEL + AlphScoreclassified variants for *TARDBP*, *FUS*, *SETX*, *TBK1*, *OPTN*, *SOD1*, *VCP*, *SQSTM1*, *ANG*, *UBQLN2*, *DCTN1*, and *CCNF* as positive and negative. For MOVA and MOVA + REVEL, the stratified fivefold cross validation was repeated 5 times, so the cvAUC function of the cvAUC package was used to draw the average of the ROC curves for 25 times.

## Data Availability

The source code used in this study is available at https://github.com/yuya-hatano/MOVA. Of the data used in this analysis, positive and negative variant data were obtained from HGMD Professional 2022.4 (https://digitalinsights.qiagen.com/products-overview/ clinical-insights-portfolio/human-gene-mutation-database/) and gnomAD v.3.1.2 (https://gnomad.broadinstitute.org/). AlphScore_final.tsv was obtained from https://zenodo.org/record/6288139. The dbNSFP4.3a file was obtained from http://database.liulab.science/dbNSFP#version. The amino acid sequence of the target protein was obtained from https://asia.ensembl.org/index.html. The predicted conformation data predicted by AlphaFold2 was obtained from https://alphafold.ebi.ac.uk/.

## Abbreviations

ALS: Amyotrophic lateral sclerosis
MOVA: Method for evaluating the pathogenicity of missense variants using AlphaFold2
FALS: Familial amyotrophic lateral sclerosis
SALS: Sporadic amyotrophic lateral sclerosis
3D: Three-dimensional
ACMG: The American College of Medical Genetics and Genomics
AMP: The Association for Molecular Pathology
pLDDT: Predicted local distance difference test
SVM: Support vector machine
5F-MV value: 5-Fold MOVA value
IDRs: Intrinsic disordered regions
ROC: Receiver operating characteristic
