# Supplementary figures and images for: Accuracy of a machine learning method based on structural and locational information from AlphaFold2 for predicting the pathogenicity of TARDBP and FUS gene variants in ALS
Source: BMC Bioinformatics. 2023 May 19;24:206. doi: 10.1186/s12859-023-05338-5 (PMC10197232; doi:10.1186/s12859-023-05338-5)

TARDBP

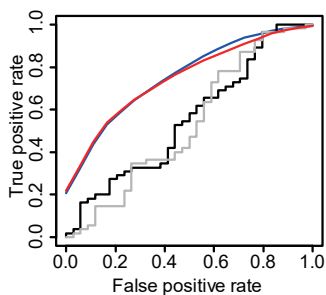

FUS

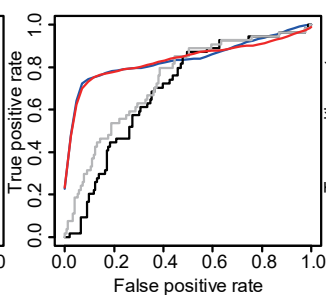

SETX

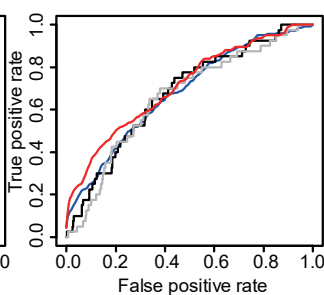

CCNF

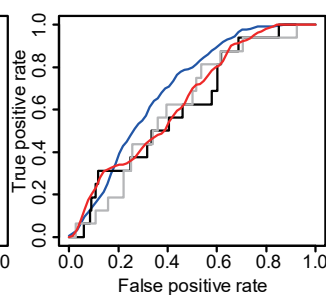

TBK1

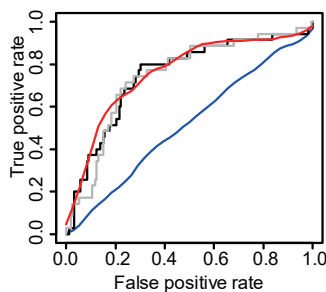

OPTN

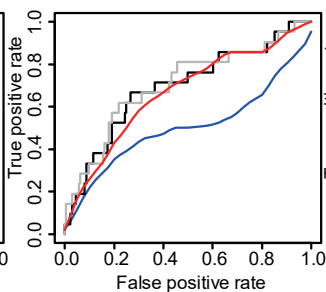

SOD1

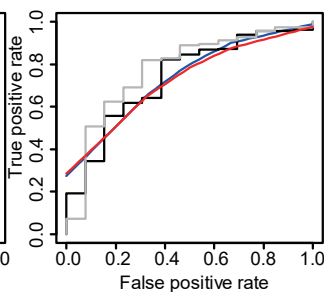

DCTN1

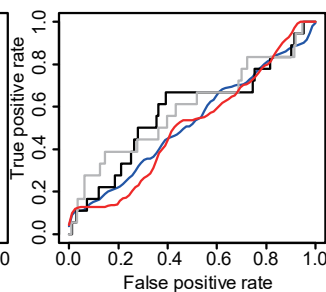

VCP

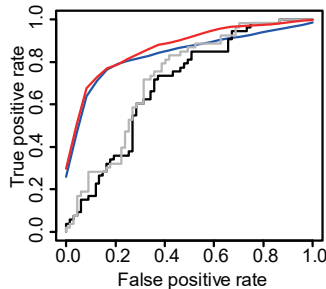

SQSTM1

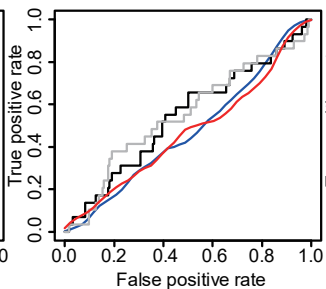

ANG

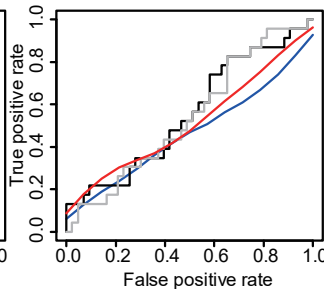

UBQLN2

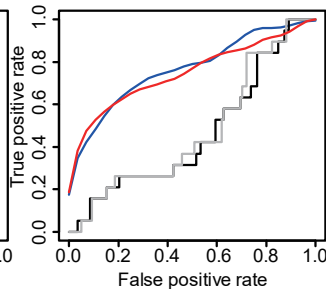

— MOVA+CADD — MOVA — CADD — CADD+AlphaScore

Supplement: Supplementary file 17 — Additional file 17: Figure S1. We used receiver operating characteristiccurve analysis to determine whether MOVA + CADD, MOVA, CADD, or CADD + AlphScoreclassified variants for TARDBP, FUS, SETX, TBK1, OPTN, SOD1, VCP, SQSTM1, ANG, UBQLN2, DCTN1, and CCNF as positive and negative. For MOVA and MOVA + CADD, the stratified fivefold cross validation was repeated 5 times, so the cvAUC function of the cvAUC package was used to draw the average of the ROC curves for 25 times. [file 12859_2023_5338_MOESM17_ESM.pdf]
