# Supplementary material for: Accuracy of a machine learning method based on structural and locational information from AlphaFold2 for predicting the pathogenicity of TARDBP and FUS gene variants in ALS
Source: BMC Bioinformatics. 2023 May 19;24:206. doi: 10.1186/s12859-023-05338-5 (PMC10197232; doi:10.1186/s12859-023-05338-5)
Supplement: Supplementary file 18 — Additional file 18: Figure S2. We used receiver operating characteristiccurve analysis to determine whether MOVA + REVEL, MOVA, REVEL, or REVEL + AlphScoreclassified variants for TARDBP, FUS, SETX, TBK1, OPTN, SOD1, VCP, SQSTM1, ANG, UBQLN2, DCTN1, and CCNF as positive and negative. For MOVA and MOVA + REVEL, the stratified fivefold cross validation was repeated 5 times, so the cvAUC function of the cvAUC package was used to draw the average of the ROC curves for 25 times. [file 12859_2023_5338_MOESM18_ESM.pdf]

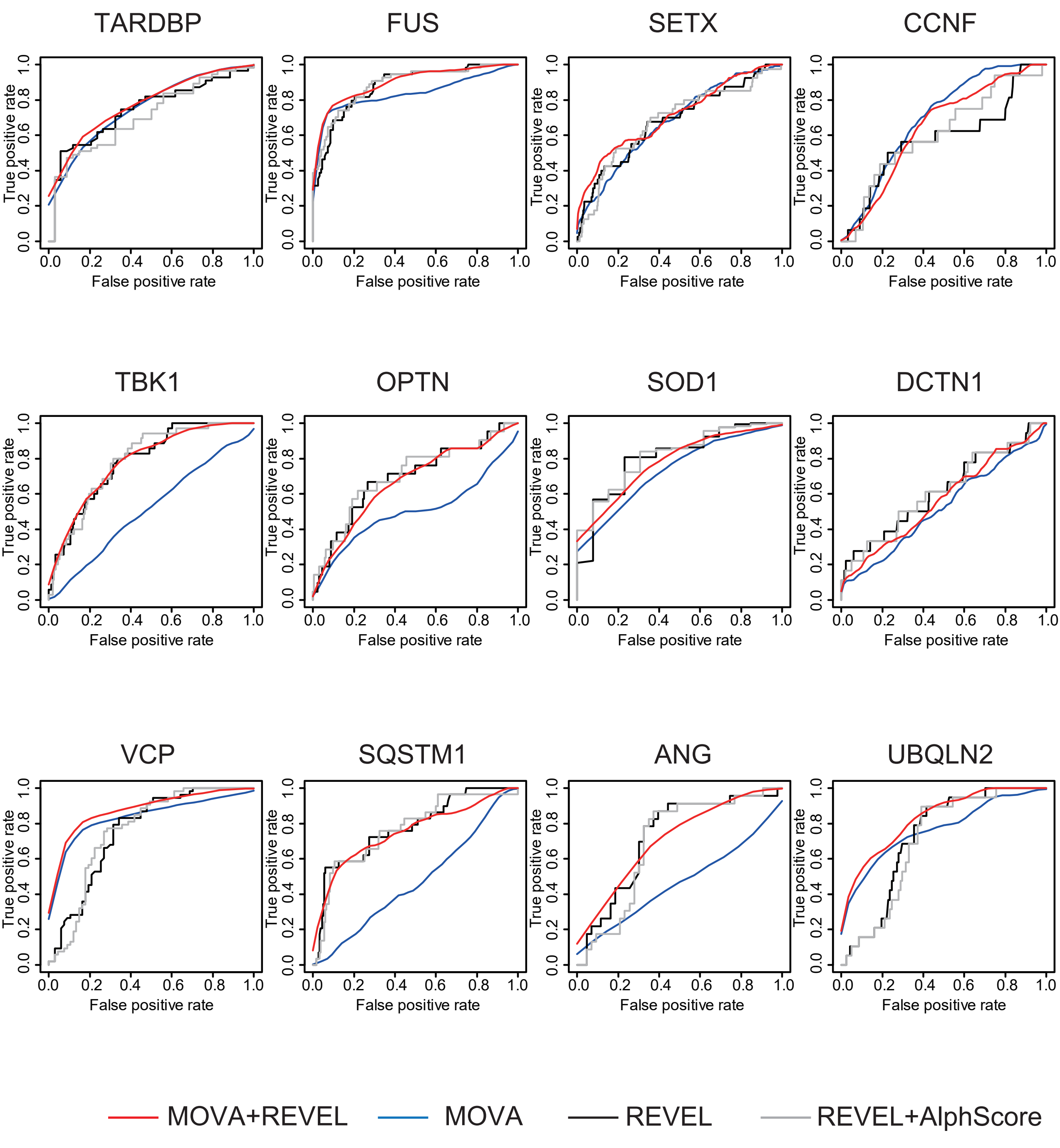
TARDBP
FUS
SETX
CCNF
TBK1
OPTN
SOD1
DCTN1
VCP
SQSTM1
ANG
UBQLN2
True positive rate
False positive rate
0.0
0.2
0.4
0.6
0.8
1.0
MOVA+REVEL
MOVA
REVEL
REVEL+AlphScore
